# Genetic Determinants of Receptor-Binding Preference and Zoonotic Potential of H9N2 Avian Influenza Viruses

**DOI:** 10.1128/JVI.01651-20

**Published:** 2021-02-10

**Authors:** Thomas P. Peacock, Joshua E. Sealy, William T. Harvey, Donald J. Benton, Richard Reeve, Munir Iqbal

**Affiliations:** a Department of Infectious Diseases, Imperial College London, London, United Kingdom; b Avian Influenza Group, The Pirbright Institute, Woking, United Kingdom; c Royal Veterinary College, University of London, London, United Kingdom; d Boyd Orr Centre for Population and Ecosystem Health, Institute of Biodiversity, Animal Health and Comparative Medicine, College of Medical, Veterinary and Life Sciences, University of Glasgow, Glasgow, United Kingdom; e The Francis Crick Institute, London, United Kingdom; St. Jude Children’s Research Hospital

**Keywords:** avian influenza, avian viruses, influenza, receptor binding, zoonotic infections

## Abstract

Receptor recognition and binding is the first step of viral infection and a key determinant of host specificity. The inability of avian influenza viruses to effectively bind human-like sialylated receptors is a major impediment to their efficient transmission in humans and pandemic capacity. Influenza H9N2 viruses are endemic in poultry across Asia and parts of Africa, where they occasionally infect humans and are therefore considered viruses with zoonotic potential. We previously described H9N2 viruses, including several isolated from human zoonotic cases, which showed a preference for human-like receptors. Here, we take a mutagenesis approach, making viruses with single or multiple substitutions in H9 hemagglutinin and testing binding to avian and human receptor analogues using biolayer interferometry. We determine the genetic basis of preferences for alternative avian receptors and for human-like receptors, describing amino acid motifs at positions 190, 226, and 227 that play a major role in determining receptor specificity, and several other residues such as 159, 188, 193, 196, 198, and 225 that play a smaller role. Furthermore, we show that changes at residues 135, 137, 147, 157, 158, 184, 188, and 192 can also modulate virus receptor avidity, and substitutions that increased or decreased the net positive charge around the hemagglutinin receptor-binding site show increases and decreases in avidity, respectively. The motifs we identify as increasing preference for the human-like receptor will help guide future H9N2 surveillance efforts and facilitate our understanding of the emergence of influenza viruses with increased zoonotic potential.

**IMPORTANCE** As of 2020, over 60 infections of humans by H9N2 influenza viruses have been recorded in countries where the virus is endemic. Avian-like cellular receptors are the primary target for these viruses. However, given that human infections have been detected on an almost monthly basis since 2015, there may be a capacity for H9N2 viruses to evolve and gain the ability to target human-like cellular receptors. Here, we identify molecular signatures that can cause viruses to bind human-like receptors, and we identify the molecular basis for the distinctive preference for sulfated receptors displayed by the majority of recent H9N2 viruses. This work will help guide future surveillance by providing markers that signify the emergence of viruses with enhanced zoonotic potential, as well as improving understanding of the basis of influenza virus receptor binding.

## Abstract (precis)

As of 2020, over 60 infections of humans by H9N2 influenza viruses have been recorded in countries where the virus is endemic. Avian-like cellular receptors are the primary target for these viruses.

## INTRODUCTION

In the 1990s, avian influenza virus subtypes H5N1 and H9N2 underwent a host switch from wild birds to domestic poultry, where they have circulated ever since. H9N2 has since become one of the most widespread strains in poultry, infecting domestic fowl throughout Asia and North Africa, where it circulates hyper-endemically (1–4). Zoonotic H9N2 cases are also occasionally detected, with human infections reported in Hong Kong, mainland China, Bangladesh, Egypt, Pakistan, Oman, India, and Senegal; over half of human infections have been reported in the last 4 years alone, all of which indicates a growing pandemic threat from these viruses (4–12). Although no human-to-human transmission has been recorded, some H9N2 virus strains have shown a high propensity for airborne transmission between ferrets (13, 14), the most commonly used model for human influenza transmission.

The hemagglutinin (HA) glycoprotein mediates attachment of influenza viruses to host cells through binding of glycans with terminal sialic acid moieties. The human upper respiratory tract (URT) is rich in glycans with terminal α2,6-linked sialic acid (SA) and is the primary site of replication for human influenza viruses. An important determinant for adaptation to the human URT is the ability of HA to bind α2,6-linked sialylated glycans (15). However, most avian influenza viruses preferentially bind to glycans with terminal α2,3-linked SAs, which are common in the avian gastrointestinal and respiratory tracts (16). Therefore, for avian influenza viruses to be able to efficiently infect and transmit between humans, they must gain the ability to bind to α2,6-linked SA. Furthermore, contemporary chicken-adapted H9N2 and H6N1 (Taiwanese lineage) viruses have specifically been identified as having a receptor preference for α2,3-linked SA, sulfated on the antepenultimate sugar, while highly pathogenic H5 and H7, as well as low-pathogenicity avian influenzas viruses from waterfowl, appear to bind sulfated and nonsulfated α2,3-linked SA equally well (17–25).

Several molecular determinants have been shown to influence the receptor-binding profile of H9N2 viruses, including position 226 (H3 HA numbering used throughout; position 216 in mature polypeptide H9 numbering); in one strain, the Q226L substitution alone could facilitate greater replication of an H9N2 isolate in human epithelial airway cells (26). Substitutions at 155, 190, and 227 (145, 180, and 217 in H9 numbering) have also been shown to play a role in receptor-binding preference in some H9N2 viruses (13, 17, 27, 28). However, understanding of H9N2 receptor-binding preference remains piecemeal, with no studies having systematically looked at the roles of multiple residues singly and in combinations.

In a previous study, we described notable variability in receptor-binding preference among circulating H9N2 viruses, which we hypothesized was due to amino acid variability at residues 190, 226, and 227 (18). Here, we take three H9N2 viruses representative of different receptor-binding profiles, including a virus isolated from a human with a natural preference for α2,6-linked SA, and generate recombinant virus libraries with HA amino acid substitutions that represent reciprocal changes at each nonconserved amino acid position in the vicinity of the receptor-binding site between the progenitor H9N2 viruses. We test the receptor binding ability of these libraries using biolayer interferometry (BLI) and show that changes at residues 190, 226, 227, and, to a lesser extent, 159, 188, 193, 196, 198, and 225, explain this receptor preference variability. We further show that several antibody escape mutants have changed receptor preference or avidity, and we describe a correlation between the electrostatic charge of the HA head and receptor avidity. Finally, we use the insights from these experiments to predict H9N2 lineages within the general viral population that have enhanced propensity to bind human receptors, and therefore possess a higher zoonotic potential.

## RESULTS

**FIG 1:**
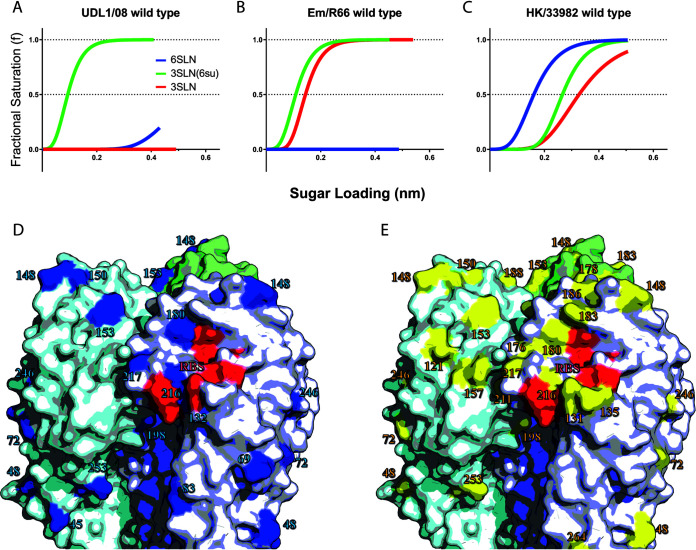
Various receptor-binding profiles of naturally occurring viruses. Biolayer interferometry was used to determine the receptor-binding profiles of H9N2 viruses UDL1/08 (A), Em/R66 (B), and HK/33982 (C). Binding was measured to three receptor analogues representative of: (i) an avian-like receptor (α2,3-sialyllactosamine or 3SLN, red lines), (ii) a sulfated avian-like receptor (Neu5Ac α2,3 Gal β1-4(6-HSO3)GlcNAc or 3SLN(6su), green lines), and (iii) a human-like receptor (α2,6-sialyllactosamine or 6SLN, blue lines). Where no binding was observed, the lines are omitted. Sequence differences in HA between UDL1/08 and Em/R66 (D) are shown in blue and between UDL1/08 and HK/33982 (E) are shown in yellow. Selected receptor-binding residues are shown in red. The figure uses the structure of PDBID 1JSH (44) and was made using PyMol (45).

Three previously characterized H9N2 viruses were chosen to act as mutagenesis backgrounds due to their distinct receptor-binding phenotypes (18). Receptor-binding profiles were measured using BLI with three receptor analogues: sulfated and nonsulfated 3′sialyllactosamine (3SLN[6su] and 3SLN, respectively) and 6′sialyllactosamine (6SLN). Both 3SLN(6su) and 3SLN are analogues for avian-like receptors, while 6SLN is an analogue for human-like receptors. The virus A/chicken/Pakistan/UDL-01/2008 (UDL1/08) displays high binding avidity to 3SLN(6su), but not 3SLN (avian-like), with residual binding to the human-like receptor 6SLN (Fig. 1A), similar to the majority of contemporary H9N2 viruses (17–19). The virus A/chicken/Emirates/R66/2002 (Em/R66) binds to both 3SLN(6su) and 3SLN but has no detectable binding to 6SLN (Fig. 1B), similar to conventional avian-adapted H5N1 and H7 viruses (18, 23). Finally, A/Hong Kong/33982/2009 (HK/33982) binds to all three receptor analogues, but with an appreciable preference for human-like 6SLN, similar to early human pandemic H3N2 viruses and zoonotic H7N9 viruses (Fig. 1C) (18, 24). To test the molecular basis of these different receptor preferences, libraries of individual or multiple reciprocal mutants were generated between these viruses with a particular focus on positions 190, 226, and 227, as well as several other nearby receptor-binding site (RBS) residues.

### Molecular basis of preference for sulfated and nonsulfated avian receptors.

To explore the molecular basis of the preference of many contemporary H9N2 strains for sulfated avian receptors, a property not shared with non-H9N2 avian influenza viruses, we investigated amino acid differences between UDL1/08 and Em/R66, which do and do not show this preference, respectively (17, 18, 20). Generally, these viruses had few differences near the RBS, though they did differ at the positions 190, 226, and 227, to which substitutions were introduced singly and in combination (Fig. 1D).

**FIG 2:**
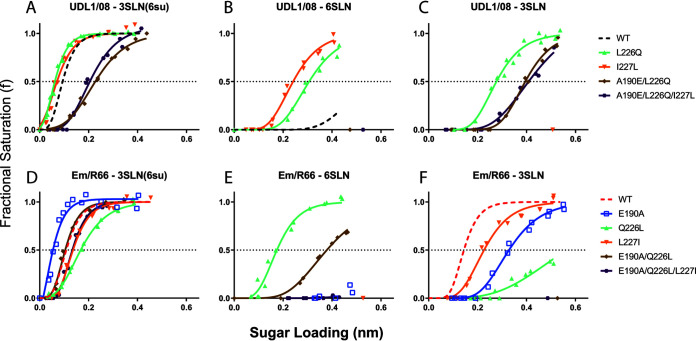
Receptor-binding profiles of reciprocal UDL1/08-Em/R66 mutants. Biolayer interferometry was used to determine the receptor-binding profiles of mutant UDL1/08 (A to C; UDL1/08 A190E could not be rescued) and Em/R66 viruses (D to F). Binding was measured to three receptor analogues: (i) Neu5Ac α2,3 Gal β1-4(6-HSO3)GlcNAc (3SLN[6su]) (A and D), (ii) α2,6-sialyllactosamine (6SLN) (B and E); and (iii) α2,3-sialyllactosamine (3SLN) (C and F). Dashed black and red lines show wild-type UDL1/08 and Em/R66 binding, respectively. The figure summarizes the data in Table 3.

Substituting all three variable RBS residues (190/226/227) led to an approximate switch of the receptor-binding phenotypes. The triple substitution increased UDL1/08 binding to 3SLN and eliminated binding to 6SLN, while Em/R66 abolished 3SLN binding and increased 6SLN binding (Fig. 2, purple lines). Generally, 190/226 reciprocal mutants expressed similar phenotypes to the triple mutants (Fig. 2, brown lines), suggesting L227I and I227L had only a modest influence. The exception to this was that Em/R66 E190A/Q226L/L227I did not show the increased binding to 6SLN observed with Em/R66 E190A/Q226L (Fig. 1E). However, in each case the exact binding avidities to the three receptor analogues were not completely recapitulated, i.e., both triple mutants had reduced avidity compared to their wild-type parental viruses, indicating that further substitutions are required to fully recover the receptor-binding profile of the donor viruses. Nonetheless, positions 190, 226, and, to a lesser extent, 227 appeared to be the primary determinants of variation in receptor-binding preference phenotypes of UDL1/08 and Em/R66 (Table 1).

**TABLE 1:** Hemagglutinin amino acid differences modulating preference for sulfated (3SLN[6su]) and nonsulfated (3SLN) avian-like receptors

Residue (H3 no.)	Residue (H9 no.)	WT virus	Substitution	Avian-like receptor^*a*^		Human-like receptor
				3SLN(6su)	3SLN	6SLN
190	180	UDL1/08	A to E	DNR	DNR	DNR
		Em/R66	E to A	+ +	− −	null
226	216	UDL1/08	L to Q	+	++	++
		Em/R66	Q to L	−	− − −	+++
227	217	UDL1/08	I to L	+	null	++
		Em/R66	L to I	−	−	null
190/226	180/216	UDL1/08	A to E, L to Q	− −	+	−
		Em/R66	E to A, Q to L	=	− − −	+
190/226/227	180/216/217	UDL1/08	A to E, L to Q, I to L	− −	+	−
		Em/R66	E to A, Q to L, L to I	−	− − −	null

a Symbols: = indicates <2-fold difference; + or − indicates a 2- to 10-fold increase/decrease; ++ or − − indicates a 10- to 100-fold increase/decrease; +++ or − − − indicates a >100-fold increase/decrease in binding relative to the wild-type virus; null, no difference was able to be seen because no binding to this analogue was detected with or without the substitution. DNR, indicates the virus was unable to be rescued.

Considering single residues, substitutions at position 190 exerted a strong influence on the preference for sulfated or nonsulfated avian-like receptors (3SLN[6su] and 3SLN, respectively), where viruses with A190 showed increased binding to 3SLN(6su) and decreased binding to nonsulfated 3SLN analogues, while those with E190 showed the opposite (Fig. 2A, C, D, and F). This is potentially due to a charge repulsion between the negatively charged side chain of glutamate and the sulfate group of 3SLN(6su). Additionally, viruses containing A190 generally retained or had enhanced human-like 6SLN binding, as seen in UDL1/08 wild type and the mutants Em/R66 E190A and Em/R66 E190A/Q226L (Fig. 2B and E). This was also exemplified in the differences in binding between UDL1/08 mutants; A190E/L226Q led to a loss of 6SLN binding while L226Q alone did not (Fig. 1B, mint and brown lines).

At position 226, we determined that substitutions were exerting an effect on receptor-binding avidity, consistent with the results of our previous work with erythrocyte-based avidity assays (29). Viruses with Q226 bound with higher avidity, compared with L226, to each of the three analogues tested (Fig. 2A to F). Additionally, Q226 favored 3SLN binding, as can be seen by the mutant UDL1/08 L226Q, and the difference in avidity between Em/R66 E190A/Q226L and E190A alone (Fig. 2C and F, blue and brown lines). In the background of Em/R66, Q226L showed a large increase in 6SLN binding (Fig. 2E, mint line); however, a matching reduction in 6SLN binding by UDL1/08 L226Q was not seen (Fig. 2B), indicating this is probably dependent on the context of the other amino acids in the H9 HA RBS, as we had previously predicted (18).

Finally, substitutions at position 227 were identified as playing a minor role in the modulation of avidity, though they did not change receptor preference or regulate complete gain or loss of binding to any analogue. Relative to the parental virus, UDL1/08 I227L showed higher avidity to all receptors while Em/R66 L227I showed lower avidity (Fig. 2, orange lines), consistent with previous inferences made from indirect avidity measurements (29).

### Molecular basis of preference for the human receptor.

We next investigated the molecular basis of human-like receptor-binding expressed by some H9N2 viruses. A reciprocal library was generated between UDL1/08, which expresses a preference for the sulfated avian-like receptor (3SLN[6su]), and HK/33982, a virus from a human H9N2 case that binds strongly to the human-like receptor (6SLN) and has moderate binding to both avian-like receptors (Fig. 1). The amino acids at the three residues (190, 226, and 227) discussed above largely determine preference for sulfated versus nonsulfated 3SLN, and also vary between these two viruses. In addition to these three positions, reciprocal mutations were introduced at positions 188 and 193 (178 and 183 in the H9 numbering) on the basis that these residues are located next to the binding site and vary between UDL1/08 and HK/33982 (Fig. 1E).

**FIG 3:**
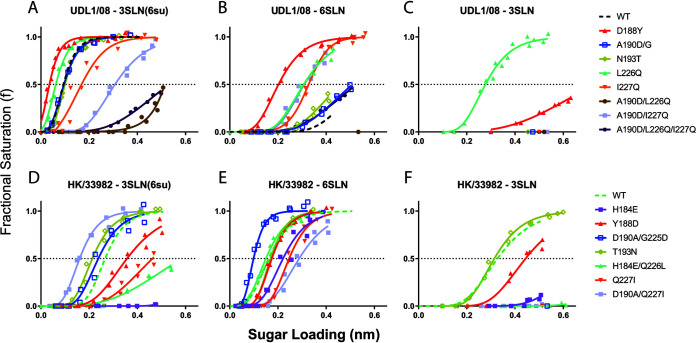
Receptor-binding profiles of reciprocal UDL1/08-HK/33982 mutants. Biolayer interferometry was used to determine the receptor-binding profiles of mutant UDL1/08 (A to C) and HK/33982 viruses (D to F). Binding was measured to three receptor analogues: (i) Neu5Ac α2,3 Gal β1-4(6-HSO3)GlcNAc (3SLN[6su]) (A and D), (ii) α2,6-sialyllactosamine (6SLN) (B and E); and (iii) α2,3-sialyllactosamine (3SLN) (C and F). Dashed black and green lines show wild-type UDL1/08 and HK/33982 binding, respectively. The figure summarizes the data in Table 4.

Introducing the substitutions A190D, L226Q, and I227Q from HK/33982 into UDL1/08 led to a loss of most of its 3SLN(6su) binding and a slight gain in 6SLN binding (Fig. 3A and B, purple lines); however, this binding phenotype did not resemble that of HK/33982. This suggests additional substitutions are required for a full gain of the human-adapted receptor-binding profile. The reciprocal triple mutant was unable to be recovered in the HK/33982 background, indicating that additional residues must play a role in stabilizing residues 190, 226, and 227 between these two viruses (Table 2).

**TABLE 2:** Attempted mutants that were not rescued or that were only rescued with compensatory changes or additional directed mutagenesis or showed a reversion

Attempted mutant	Rescued?	Compensatory change^*a*^	Additional substitution needed	Reverted
UDL1/08 A190E	No	NA	Yes, L226Q, I227I, or I227Q	NA
UDL1/08 A190D	Yes	NA	NA	Yes, D190G (mixed, equal)
HK/33982 D190A	Yes	Yes, G225D (total change)	NA	NA
HK/33982 Q226L	Yes	Yes, H184E (total change)	NA	NA
HK/33982 G225D	No	NA	Yes, D190A	NA
HK/33982 D190A/Q226L	No	NA	NA	NA
HK/33982 D190A/Q226L/Q227I	No	NA	NA	NA

a NA, not applicable; total change indicates presence of singlet on.

Introduction of reciprocal substitutions at residues 190 and 227 together indicated that these positions likely play an important role in modulation of preference between the 3SLN(6su) and 6SLN receptor analogues. UDL1/08 with A190D/I227Q showed slightly decreased binding to 3SLN(6su) and appreciably increased binding to 6SLN (Fig. 3A and B, gray lines). HK/33982 D190A/Q227I had reduced receptor-binding to 6SLN and increased binding to 3SLN(6su), creating an approximation of the receptor-binding preference of wild-type UDL1/08 (Fig. 3D and E, gray lines). From this mutant library it is clear that switches at position 226 create an incompatibility in both virus backgrounds, with several viruses losing nearly all receptor binding (UDL1/08 A190D/L226Q and A190D/L226Q/I227Q) or infectious virus being unable to be rescued entirely (HK/33982 D190A/Q226L and D190A/Q226L/Q227I; see Table 2). This was consistent with our previous work, where we saw a number of reciprocal mutants in HA required compensatory changes or else mutants rapidly reverted upon successful rescue (29).

Concerning single amino acid substitutions, amino acid substitutions at residue 190 in the single reciprocal mutants of UDL1/08 and HK/33982 showed potential incompatibilities. UDL1/08 A190D showed a mixed population also including 190G, while HK/33982 D190A gained the additional substitution G225D, as previously described (29). HK/33982 D190A/G225D increased 6SLN binding and 3SLN(6su) binding while decreasing 3SLN binding, similar to Em/R66 E190A (Fig. 3D to F, blue lines). However, HK/33982 with the G225D substitution alone was also unable to produce infectious virus, complicating interpretation of the influence of these mutations. UDL1/08 A190D/G showed no difference in binding compared to UDL1/08 (Fig. 2A to C, blue lines); however, it is difficult to interpret these data, given the mixed population.

Similar to the reciprocal mutants of UDL1/08 and Em/R66, amino acid substitutions at residue 226 between UDL1/08 and HK/33982 displayed a clear avidity effect, with 226L appearing to confer higher avidity compared with 226Q. HK/33982 with Q226L gained the additional compensatory substitution H184E (Table 2). HK/33982 with H184E alone showed a large reduction in avidity to all receptor analogues, whereas HK/33982 with H184E/Q226L increased avidity to both 6SLN and 3SLN(6su) relative to H184E alone, suggesting Q226L increased binding avidity (Fig. 3D to F, purple and mint lines; Table 3). The mutant HK/33982 H184E/Q226L also showed a strong preference for 6SLN.

**TABLE 3:** Hemagglutinin amino acid differences modulating preference for avian and human-like receptors^*a*^

Residue (H3 no.)	Residue (H9 no.)	WT virus	Substitution	Avian-like receptor		Human-like receptor
				3SLN(6su)	3SLN	6SLN
184	174	HK/33982	H to E	− − −	− −	−
188	178	UDL1/08	D toY	+++	+	++
		HK/33982	Y to D	−	−	=
190	180	UDL1/08	A to D/G	=	null	=
		HK/33982	D to A	DNR	DNR	DNR
193	183	UDL1/08	N to T	=	null	+
		HK/33982	T to N	+	=	=
196	186	UDL1/08	T to K	− −	null	+ +
225	215	HK/33982	G to D	DNR	DNR	DNR
226	216	UDL1/08	L to Q	+	++	++
227	217	UDL1/08	I to Q	=	null	+ +
		HK/33982	Q to I	− −	− − −	−
184/226	174/216	HK/33982	Q to L, H to E	− −	− − −	=
190/225	180/215	HK/33982	D to A, G to D	=	− − −	+
190/226	180/216	UDL1/08	A to D, L to Q	− − −	null	−
		HK/33982	D to A, Q to L	DNR	DNR	DNR
190/227	180/217	UDL1/08	A to D, I to Q	− − −	null	+ +
		HK/33982	D to A, Q to I	+ +	− −	− −
190/226/227	180/216/217	UDL1/08	A to D, L to Q, I to Q	− − −	null	=
		HK/33982	D to A, Q to L, Q to I	DNR	DNR	DNR

a Symbols: = indicates <2-fold difference; + or − indicates 2- to 10-fold increase/decrease; ++ or − − indicates 10- to 100-fold increase/decrease; +++ or − − − indicates >100-fold increase/decrease in binding relative to the wild-type virus; null, no difference was able to be seen because no binding to this analogue was detected with or without the substitution. DNR, indicates the virus was unable to be rescued.

At position 227, the virus UDL1/08 I227Q showed a large increase in 6SLN binding, a drop in binding to UDL1/08’s preferred receptor 3SLN(6su), and did not show altered binding to 3SLN (Fig. 3A to C, orange lines). The reciprocal mutant, HK/33982 Q227I, showed a general avidity effect with lower binding to all analogues (Fig. 3D to F, orange line). When introduced alongside D190A, the impact of Q227I in the HK/33982 D190A/Q227I double mutant reduced 6SLN binding and slightly increased binding to 3SLN(6su) relative to the parental wild type and the D190A(+G225D) mutant (Fig. 3D to F, gray and orange lines).

Reciprocal substitutions at position 188 influenced receptor binding, while those at position 193 did not. These residues have not previously been described as affecting receptor binding in H9 HA, though they are adjacent to the RBS and 193 has been described as playing a vital role in the modulation of binding of sulfated 3SLN by H5 and H7 HA (30, 31). Viruses with Y188 showed higher avidity relative to viruses with D188, regardless of the virus background (Fig. 3, red lines). Additionally, HK/33982 Y188D appeared to have minor effects on specific receptor analogue preference, with a greater reduction in 3SLN binding compared with 6SLN. Amino acid swaps at position 193 showed a very minor, nonreciprocal receptor preference effect (Fig. 3).

### Molecular basis of variation in receptor-binding avidity.

In addition to showing preference for different receptors, influenza viruses vary in receptor-binding avidity. In previous studies we have inferred that escape mutants with the largest impact of polyclonal antisera binding may be driven by avidity effects (17, 29, 32). To further investigate the genetic basis of variation in receptor-binding avidity we constructed a library of mutants in the UDL1/08 background, many of which we had previously identified as modulating virus antigenicity (29), and assessed their receptor-binding phenotypes. To complement this, we analyzed a large data set of hemagglutination inhibition (HI) titers and HA sequences from natural H9N2 viruses to identify amino acid variants correlating with avidity effects apparent in measured titers.

**FIG 4:**
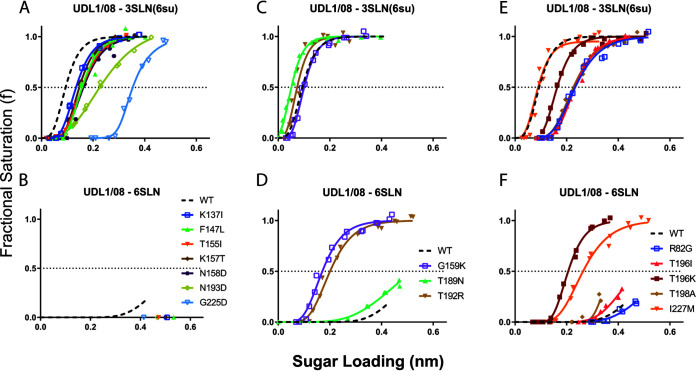
Receptor binding modulation by nonreciprocal mutations in the background of UDL1/08. Biolayer interferometry was used to determine the receptor-binding profiles of each mutant virus. Binding was measured to three receptor analogues, α2,6-sialyllactosamine (6SLN), α2,3-sialyllactosamine (3SLN), and Neu5Ac α2,3 Gal β1-4(6-HSO3)GlcNAc (3SLN[6su]). Dashed black lines show wild-type UDL1/08 binding. Panels indicate binding by mutants that show an avidity reduction (A and B), an increase in avidity (C and D), and changes in receptor-binding preference (E and F). No mutants had any detectable binding to 3SLN.

Testing of UDL1/08 mutants showed several exhibited a general decrease in avidity to the tested analogues, including K137I, F147L, K157T, N158D, N193D, and G225D (Fig. 4A and B). A single substitution, T189N, showed a negligible effect on receptor binding (Fig. 4C and D, mint lines). Two substitutions, T135K and T192R, appeared to increase avidity (i.e., increased binding to all analogues tested) in a similar manner to UDL1/08 I227L and D188Y from the reciprocal mutant libraries (Fig. 4C and D, Fig. 2A to C, and Fig. 3A to C). The substitution R82G showed a reduction in 3SLN(6su) binding without affecting 6SLN binding (Fig. 4E and F, blue lines). Finally, one group of mutants showed changes in receptor-binding preference with increases in human-like 6SLN binding relative to sulfated avian-like receptor 3SLN(6su): (i) I227M showed a large increase in 6SLN binding without changing 3SLN(6su) binding (orange lines); (ii) G159K showed a modest increase in avidity to 3SLN(6su) but a much larger relative increase in 6SLN binding (purple lines); and (iii) T196I, T196K, and T198A all decreased 3SLN(6su) binding while increasing 6SLN binding to various degrees (Fig. 4E and F, light red, dark red, and light brown lines). This last group of mutations represents single amino acid changes that could act as markers for viruses with greater zoonotic potential, along with the previously described Q226L (in the background of Em/R66 and HK/33982) and I227Q in a UDL1/08-like background (Fig. 2E, Fig. 3B and E).

**FIG 5:**
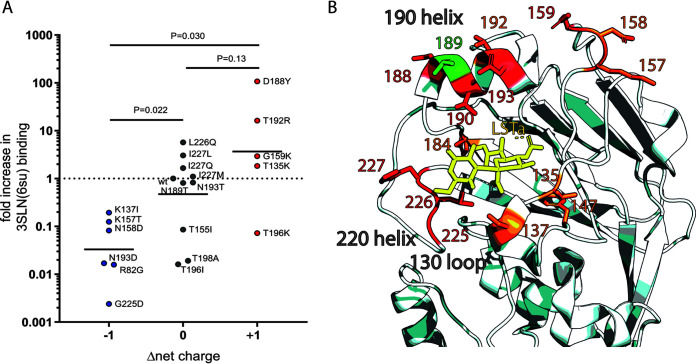
Mapping of residues tested for receptor binding changes and correlation between charge and 3SLN(6su) binding. (A) The fold increase in binding to 3SLN(6su) is plotted along with the introduced change in net charge difference. Wild-type UDL1/08 binding to 3SLN(6su) is set to 1, with relative binding calculated as described previously (18). Annotated *P* values were calculated using a nonparametric Mann-Whitney U test, where lines indicate mean fold increases. (B) Structure schematic. Residues which affect receptor-binding preference (i.e., affect binding to different analogues in different ways) are shown in red; residues that affect receptor-binding avidity across analogues are shown in orange. Residues that when mutated have no effect on receptor binding are shown in green. LSTa, shown in yellow, is an α2,3-linked (avian-like) receptor analogue. H3 HA numbering was used throughout. The figure uses the structure of PDBID 1JSH (44) and was made using PyMol (45).

In general, amino acid replacements that increased positive charge in the HA head domain tended to cause an increase in receptor-binding avidity, while the opposite was true for substitutions that increased negative charge. In Fig. 5A, the impact of substitutions introduced to the UDL1/08 backbone on avidity for its preferred receptor, 3SLN(6su), is plotted by the change in net charge of the HA head domain. The proximity of the residues at which substitutions were introduced to the RBS is shown in Fig. 5B.

To investigate variation in avidity among natural H9N2 viruses, we modeled variation in HI titers for a data set of a large number of viruses covering all major H9 lineages. In addition to measuring antigenic relationships, HI titers are influenced by avidity. Viruses with higher avidity require more antibodies to inhibit hemagglutination, manifesting as a tendency toward lower HI titers regardless of antigenic relationships to the antisera used, and vice versa. To identify amino acid variants correlating with such variation in titers, we adapted a model we previously developed to identify molecular determinants of antigenic variation in both human and avian influenza viruses (29, 33).

Variation in HI titers resulting from antigenic differences and from variation in virus avidity was mapped to branches of the HA phylogenetic tree as previously described (29). To explore the genetic basis of variation caused by differences in avidity, phylogenetic terms representing branches leading to clades of viruses with systematically higher or lower titers were replaced with terms representing amino acid identity in the test virus at each variable HA position in turn. Under a forward selection procedure, amino acids at positions 190, 196, and 198 were identified as contributing to variation in avidity. Each of these positions are in the 190-helix, proximal to the RBS. Position 190 has already been shown to play a role in receptor binding in this and other studies (17, 27), and we see the effect of 196 and 198 on receptor-binding preference in this study (Fig. 4E and F), further validating this modeling approach (Table 4).

**TABLE 4:** Amino acid residues predicted to explain variation in HI titers as a result differences in receptor-binding avidity

Residue (H3 no.)	Residue (H9 no.)	Distinct amino acid residues	No. of HI titers	Effect on 3SLN(6su) binding^*a*^
190	180	A	1,145	none
		T	309	50× increase
		V	494	1,400× increase
196	186	T	1,912	none
		I	331	41× decrease
		K	296	7× decrease
198	188	T	1,913	none
		A	351	41× decrease

a Effect on 3SLN(6su) binding is shown relative to most common amino acid in meta-analysis data set: 190A, 196T, and 198T.

### Sequence-based prediction of receptor-binding preference of H9N2 viruses.

**FIG 6:**
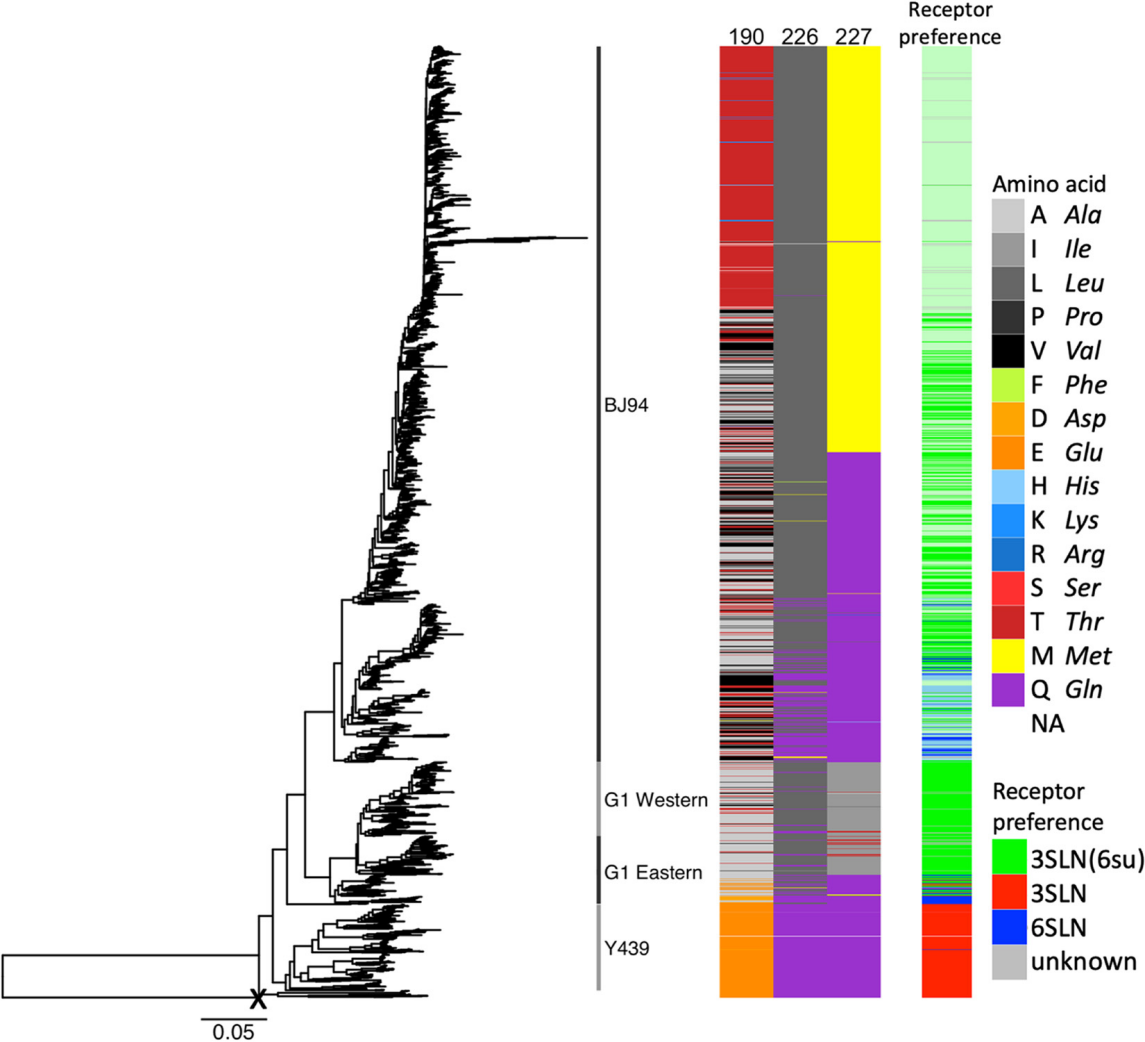
Sequence variation and predicted receptor preference across H9N2 phylogeny. HA phylogeny for 2,440 H9N2 viruses was rooted with a sequence from H9-like bat influenza virus (marked with X). The amino acid identity at positions 190, 226, and 217 is shown by color and grouped by side chain property, according to the legend. The receptor-binding preferences of viruses were predicted as being for either the sulfated avian-like receptor (green), nonspecific avian-like receptor (sulfated or nonsulfated, red), or for the human-like receptor (blue), with lighter shades indicating reduced confidence (further details in the Materials and Methods). The predicted receptor preference is based on amino acid identity at positions 190, 226, and 227 from extrapolation of testing of viruses generated in mutagenesis experiments using biolayer interferometry with a range of receptor analogues.

Finally, we extrapolated the BLI results with the 3SLN, 3SLN(6su), and 6SLN receptor analogues to predict receptor-binding preferences of circulating H9N2 viruses on the basis of amino acid identity at positions 190, 226, and 227 according to Table 5. Viruses were predicted as possessing one of three receptor-binding phenotypes: (i) a strong preference for sulfated avian-like receptors, typical of chicken-adapted H9N2 viruses; (ii) a receptor-binding phenotype more similar to chicken-adapted H5Nx or H7N1 viruses that bind both sulfated and nonsulfated avian-like receptors; or (iii) a preference for the human-like receptor with concurrent binding to avian receptors. Amino acid identity at positions 190, 226, and 227 and the resulting prediction is shown across a phylogeny constructed using all available H9N2 HA sequences in Fig. 6. Almost all viruses in the Y439-like lineage, prevalent in wild birds and in poultry in Korea, as well as a few viruses in the G1 Eastern sublineage are predicted to show an Em/R66-like preference for any avian-like receptor. The vast majority of viruses in the chicken-adapted BJ94 and G1 lineages are predicted to express a sulfated avian receptor preference. A significant number of viruses belonging to the G1 Eastern sublineage, prevalent in minor poultry in China, are predicted to show a preference for human-like receptors, as are a number of viruses interspersed within a clade of viruses belonging to the BJ94 lineage.

**TABLE 5:** Amino acid motifs across positions 190, 226, and 227 and accompanying prediction of receptor preference

H3 amino acid residue (H9 numbering)			Predicted receptor preference^*a*^
190 (180)	226 (216)	227 (217)	
A/T/V	L	I/L/M/Q	Avian-like (sulphated)
A	Q	I/T	Avian-like (sulphated)
I	Q	F	Avian-like (sulphated)
E	Q	L/Q	Avian-like (nonsulphated or sulphated)
A/D/T/V	Q	Q	Human-like
E	L	Q	Human-like

a Where alternative motifs were present or sequence data at these positions was incomplete, no prediction was made.

## DISCUSSION

Receptor binding is an important determinant of host specificity, and modification of receptor-binding properties is often a critical step in cross-species virus transmission. In this study, we have comprehensively investigated residues in and around the receptor binding site (RBS) of hemagglutinin (HA) from H9N2 viruses for their ability to influence receptor preference and avidity. We have shown that different combinations of the residues 190, 226, and 227 account for much, but not all, of the variability in H9N2 receptor binding between viruses representative of the major binding phenotypes. Furthermore, we described several other residues that have strong influences on H9N2 receptor binding preference, including positions 159, 188, 193, 196, and 198. We propose a model whereby residues in the influenza HA1 that do not directly coordinate receptor binding can play a delocalized avidity role through modulating the charge of the head domain, with increases in positive charge giving a nonspecific increase in avidity and vice versa. We hypothesize this effect may be exaggerated for H9N2 viruses where sulfated, sialylated glycans appear to be the preferred receptor owing to the greater amount of negative charge in these receptors (compared to nonsulfated, sialylated glycans). Finally, we have applied the results of this study to try and predict the receptor-binding phenotypes of different circulating H9N2 viruses as a way of predicting strains that may have a heightened zoonotic potential.

Several of the residues identified in this study have been previously described as directly or indirectly affecting H9 receptor binding, including position 155, 190, 225, 226, and 227 (13, 17, 26–28, 34, 35). In previous studies, generally only one or two of these residues have been measured in isolation for their receptor-binding effect or were tested in a nonquantitative or only semiquantitative manner. Here, we performed a comprehensive, quantitative analysis, often testing changes at residues in multiple different virus backbones and in multiple combinations to investigate strain-specific and compensatory effects. For example, the substitution Q226L has been shown several times in an older G1-Eastern lineage virus to increase binding to human-like receptors (26, 36), with a similar effect when we investigated a contemporary virus of the same lineage (i.e., HK/33982). However, in a G1-Western backbone (i.e., UDL1/08) we find a completely different effect, whereby there is a general avidity increase and an overall increase in 6SLN binding when an L226Q substitution is incorporated, highlighting the importance of the context in which a mutation occurs.

In a previous study, we inferred that several substitutions that had the largest effect on immune escape in H9N2 viruses were substitutions that affected avidity, as has been predicted for human influenza viruses (29, 37, 38). Here, we demonstrated that a wide variety of escape mutants previously shown to robustly modulate polyclonal antisera binding do indeed modulate receptor-binding avidity, indicating that elevation of avidity is a viable mechanism of immune escape for H9N2 viruses, as has been shown for human influenza viruses (37, 38). We also performed an analysis of matched genetic and antigenic data for 330 H9N2 viruses, covering each major H9 lineage (29). This analysis identified that different amino acids at positions 190, 196, and 198 contributed to variation in HI titers as a result of differing avidity, as they tended to be associated with lower or higher titers after accounting for antigenic similarity to the antisera used. We also confirmed each of these three positions play an important role in regulation of receptor-binding avidity using BLI, validating this approach and indicating that BLI results are transferable to observations based on erythrocyte binding. In addition to the important role for residue 190 in receptor preference, the substitutions T196I, T196K, and T198A all showed relative increases in human-like receptor binding. These results further support the case for using integrated modeling approaches to reanalyze large data sets and predict genotype-phenotype relationships.

We present evidence suggesting that residues in the influenza HA1 that do not directly coordinate receptor binding play a delocalized avidity role through modulating the charge of the head domain. For mutations introduced in the UDL1/08 background and measured in binding to its preferred receptor, 3SLN(6su), we found a significant trend in avidity change between substitutions that introduced a negative charge and those that introduced a positive charge. In general, substitutions observed to increase avidity tended to increase the net positive charge around the RBS (e.g., T135K, G159K, T192R, D188Y, Em/R66 E190A, and Em/R66 Q226L/E190A), while avidity-decreasing mutants usually decreased the net positive charge around the RBS (e.g., R82G, K137I, K157T, N158D, N193D, G225D, HK/33982 Y188D, and HK/33982 H184E). This effect is likely due to nonspecific charge interactions with the negatively charge sialic acid, and we hypothesize this effect may be more pronounced for H9N2 viruses because the negative charge of sulfated, sialylated glycans, which appear to be their preferred receptor, is greater due to the negatively charged sulfate group.

An important contribution of this study is the identification of several substitutions that, in one or more of our virus backbones, resulted in viruses with increased or *de novo* human-like 6SLN binding. These residues will be useful for future surveillance efforts to identify newly sequenced viruses with elevated zoonotic potential. These particular substitutions include the previously well-characterized Q226L substitution in HK/33982 or Em/R66-like backbones (but not the contemporary G1-Western UDL1/08-like backbone), as well as the newly characterized R82G, T135K, G159K, T196I, T196K, T198A, I227M, and I227Q substitutions in the UDL1/08-like backbone. Further work using whole virus in biologically relevant tissue types, such as human airway epithelial cells or lung organoids, would be desirable to fully verify our biophysical receptor-binding results. Several of these mutations are already commonly found in the field, further suggesting that H9N2 virus variants naturally circulate, with a likely heightened zoonotic potential. In particular, a significant proportion of viruses belonging to the G1-Eastern sublineage, prevalent in minor poultry in China, are predicted to show a preference for human-like receptors and thus represent a zoonotic risk. We hypothesize that the enhanced preference for the human-like receptor could be an adaptation to minor poultry, which overexpress α2,6-linked SA relative to chickens.

In conclusion, we have quantified the impact of single and multiple amino acid substitutions on receptor-binding phenotypes in the context of several H9N2 viruses with various receptor-binding preferences, identifying seven novel mutations that increase binding to the human-like receptor. We further highlight the importance of mutations that impact receptor-binding avidity. Avidity modulation has a dramatic impact on antigenicity and an equally important role in the receptor binding-phenotype, such that viruses gaining avidity-enhancing mutations may present multiple challenges, both compromising vaccine efficacy and increased zoonotic potential. As well as helping to better understand the molecular basis of avian influenza receptor binding, the results generated here will help future surveillance efforts to identify viruses which may potentially have an augmented zoonotic potential and/or greater vaccine escape potential.

## MATERIALS and METHODS

### Ethics statement.

Use of embryonated eggs in this study was carried out in strict accordance with European and United Kingdom Home Office regulations and the Animals (Scientific Procedures) Act 1986 Amendment Regulations, 2012. These studies were carried out under the United Kingdom Home Office-approved project license number P68D44CF.

### Cell lines and eggs.

HEK 293T and MDCK cells were maintained in Dulbecco’s modified Eagle medium (DMEM) supplemented with 10% fetal calf serum (FCS) at 37°C and 5% CO_2_. Viruses were propagated in 10-day-old embryonated eggs and allantoic fluid was harvested at 48 h postinoculation.

### Viruses.

Throughout this study, recombinant viruses generated by standard 8 plasmid influenza reverse genetics used HEK 293T/MDCK coculture (39). All viruses contained the named HA gene (whether wild type or mutant), the neuraminidase (NA) gene of A/chicken/Pakistan/UDL-01/2008 (UDL1/08), and the remaining genes from A/Puerto Rico/8/1934 (PR8), allowing for high viral titers from eggs. Mutant HA plasmids were generated by site-directed mutagenesis. Viruses were attempted to be rescued a minimum of three independent times and left for 7 days postcoculture before being determined to be unrescuable. All amino acid differences between the three representative strains, at HA positions in the vicinity of the receptor-binding site, were tested as mutations capable of influencing binding phenotype. A list of viruses used in this study can be found in Table 6.

**TABLE 6:** Full list of viruses rescued (or attempted) in this study

Virus background	Mutation (H3 numbering)	Mutation (H9 numbering)	Notes^*a*^
UDL1/08	R82G	R74G	
	T135K	T129K	
	K137I	K131I	
	F147L	F137L	
	T155I	T145I	
	K157T	K147T	
	N158D	N148D	
	G159K	G149K	
	D188Y	D178Y	
	N189T	N179T	
	A190E	A180E	DNR
	A190D	A180D	Partly reverted to D190G
	T192R	T182R	
	N193D	N183D	
	N193T	N183T	
	T196I	T186I	
	T196K	T186K	
	T198A	T188A	
	G225D	G215D	
	L226Q	L216Q	
	I227L	I217L	
	I227Q	I217Q	
	I227M	I217M	
	A190E/L226Q	A180E/L216Q	
	A190D/I227Q	A180D/I217Q	
	A190E/L226Q/I227L	A180E/L216Q/I217L	
	A190D/L216Q/I227Q	A180D/L216Q/I217Q	
Em/R66	E190A	E180A	
	Q226L	Q216L	
	L227I	L217I	
	E190A/Q226L	E180A/Q216L	
	E190A/L227I	E180A/L217I	
	E190A/Q226L/L227I	E180A/Q216L/L217I	
HK/33982	H184E	H174E	Compensatory mutation of Q226L
	Y188D	Y178D	
	D190A	D180A	Got compensatory mutation G225D upon rescue
	T193N	T183N	
	G225D	G215D	DNR
	Q226L	Q216L	Got compensatory mutation H184E upon rescue
	Q227I	Q217I	
	D190A/Q226L	D180A/Q216L	DNR
	D190A/Q227I	D180A/Q217I	
	D190A/Q226L/Q227I	D180A/Q216L/Q217I	DNR

a DNR indicates “did not rescue.”

### Virus sequencing.

Viruses were sequenced to confirm no reversions or additional substitutions had occurred upon production and propagation. The HA1 region of HA was sequenced for each virus as previously described (40).

### Virus purification.

Low-speed centrifugation was initially used to remove large debris from virus-containing egg allantoic fluid. Virus particles were next pelleted by ultracentrifugation at 27,000 rpm for 2 h. Virus pellets were subsequently homogenized by glass homogenizer, resuspended, and purified with a 30 to 60% sucrose gradient. The visible band containing virus was then isolated, diluted into phosphate-buffered saline (PBS), and centrifuged for another 2 h at 27,000 rpm. The final virus pellet was then resuspended in PBS with 0.01% azide. The concentrations of purified viruses were determined using a nucleoprotein enzyme-linked immunosorbent assay (ELISA) as described previously (41).

### Biolayer interferometry.

Purified virus binding to different sialylated receptor analogues was tested using an Octet RED biolayer interferometer (Pall ForteBio), as described previously (18). Receptor analogues contained 30-kDa polyacrylamide backbones conjugated to 20 mol% trisaccharides and 5 mol% biotin (Lectinity Holdings). The three analogues used in this study were α2,6-sialyllactosamine (6SLN), α-2,3-sialyllactosamine (3SLN), and Neu5Ac α-2,3Galβ1-4(6-HSO3)GlcNAc (3SLN[6su]). Sialoglycopolyemers were bound onto streptavidin-coated biosensors (Pall ForteBio) at ranges of concentrations from 0.01 to 0.55 μg/ml in HBS-EP (10 mM HEPES [pH 7.4], 150 mM NaCl, 3 mM EDTA, 0.005% Tween 20). Virus was diluted to a concentration of 100 pM in HBS-EP, 10 μM oseltamavir carboxylate (Roche), and 10 μM zanamivir (GSK). Virus association to the bound sialoglcopolymers was measured at 20°C for 30 min. Virus binding curves were normalized to fractional saturation and plotted as a function of sugar loading. Relative dissociation constants were calculated as described previously (18, 25).

### Modeling of potential receptor-binding residues.

To identify amino acid positions where substitutions correlated with differences in receptor-binding avidity apparent in HI titers, a modeling approach previously used to identify substitutions causing antigenic differences among influenza viruses was adapted (29, 33, 42, 43). Branches of the HA phylogenetic tree correlated with variation in HI titers when the branch (i) separated test virus and antisera, (ii) descended the test virus, or (iii) descended the virus used to generate antisera. These phylogenetic terms are interpreted as being associated with changes in (i) antigenicity, (ii) receptor-binding avidity, and (iii) immunogenicity, respectively. Internal branches of the phylogeny-descending clades of two or more test viruses associated with systematically higher or lower titers and containing at least one virus also used as an antisera strain were removed, effectively dropping any terms from the model that explained variation in HI associated with differences in virus avidity. In their place, terms representing amino acid identity in the assayed virus at each variable HA position were tested. At each position, these terms allowed for titers to vary according to which amino acid residue the virus possessed to account for potential differences in contributions to avidity. These position-specific terms were added to the model under a forward selection procedure until the addition of further terms ceased to improve the model, as assessed by a likelihood ratio test (*P* < 0.05) with a Holm-Bonferroni correction for multiple testing. At selected positions, effect sizes were estimated for each alternative amino acid relative to the amino acid found most commonly in the data set.

### Prediction of receptor-binding profiles from sequence.

All available HA sequences from H9 viruses were downloaded from GIASID. A phylogenetic tree was generated from aligned nucleotide sequences using MEGA. Receptor-binding profiles were predicted across the phylogeny according to amino acid identify at positions 190, 226, and 227 on the basis of the BLI results derived during this study, which in turn build upon variation at these HA positions in sequences of natural viruses isolated from different species that varied in receptor-binding preferences as determined in a previous study (18). Viruses were predicted to exhibit preference for the sulfated avian-like receptor if at positions 190-226-227 they possessed the motifs A-L-I, A-L-L, A-L-M, A-L-Q, A-Q-I, A-Q-T, I-Q-F, T-L-I, or V-L-I (or, with reduced confidence, T-L-L, T-L-M, T-L-Q, V-L-L, V-L-M, or V-L-Q), for any avian-like receptor if they possessed the motifs E-Q-L or E-Q-Q, and for the human-like receptor if they possessed A-Q-Q, D-Q-Q, or E-L-Q (or, with reduced confidence, T-Q-Q or V-Q-Q). Predictions made with reduced confidence indicate that we have not tested the exact combination of amino acids but that the prediction is consistent with other combinations barring unforeseen interactions between sites. For viruses with incomplete sequence information at positions 190, 226, and 227 or alternative motifs, no prediction was made. Substitutions at positions 196 and 198 that increased relative 6SLN binding were not included, as it is not known which other substitutions must occur to elevate 6SLN binding above binding for the avian receptor.
